# Construction of a high-density genetic map and identification of loci controlling purple sepal trait of flower head in *Brassica oleracea L. italica*

**DOI:** 10.1186/s12870-019-1831-x

**Published:** 2019-05-30

**Authors:** Huifang Yu, Jiansheng Wang, Xiaoguang Sheng, Zhenqing Zhao, Yusen Shen, Ferdinando Branca, Honghui Gu

**Affiliations:** 1 Institute of Vegetable, Zhejiang Academy of Agricultural Sciences, Hangzhou, China; 2 Department of Agriculture, Food and Environment, University of Catania, 95123 Catania, Italy

**Keywords:** Broccoli, Genetic map, QTL, Purple sepal, SLAF

## Abstract

**Background:**

Some broccoli (*Brassica oleracea* L. *italic*) accessions have purple sepals and cold weather would deepen the purple color, while the sepals of other broccoli lines are always green even in cold winter. The related locus or gene is still unknown. In this study, a high-density genetic map was constructed based on specific locus amplified fragment (SLAF) sequencing in a doubled-haploid segregation population with 127 individuals. And mapping of the purple sepal trait in flower heads based on phenotypic data collected during three seasons was performed.

**Results:**

A genetic map was constructed, which contained 6694 SLAF markers with an average sequencing depth of 81.37-fold in the maternal line, 84-fold in the paternal line, and 15.76-fold in each individual population studied. In all of the annual data recorded, three quantitative trait loci (QTLs) were identified that were all distributed within the linkage group (LG) 1. Among them, a major locus, *qPH.C01–2*, located at 36.393 cM LG1, was consistently detected in all analysis. Besides this locus, another two minor loci, *qPH.C01–4* and *qPH.C01–5*, were identified near *qPH.C01–2*, based on the phenotypic data from spring of 2018.

**Conclusion:**

The purple sepal trait could be controlled by a major single locus and two minor loci*.* The genetic map and location of the purple sepal trait of flower heads provide an important foundation for mapping other compound traits and the identification of the genes related to purple sepal trait in broccoli.

**Electronic supplementary material:**

The online version of this article (10.1186/s12870-019-1831-x) contains supplementary material, which is available to authorized users.

## Background

Flavonoid, carotenoid and betalain together with chlorophyll in plants endow them with all kinds of colors [1–5]. Purple color is the pigment display of betalain or anthocyanin (one kind of Flavonoid). Betalain is a tyrosine-derived red-purple and yellow pigments which exist exclusively in Caryophyllales [2, 6], while anthocyanin is an abundant pigment in many different plant species and they can change color from red to purple and blue [4]. In *Brassica* plants, purple color which was caused by anthocyanin accumulation was associated with the induction of its biosynthesis genes or transcription factors [7–11]. The mutation in the upstream regulatory region of R2R3 MYB transcription factor led to increased expression levels of the transcription factor gene, which then up regulated the expression of the structural genes involved in anthocyanin biosynthesis and endowed cauliflower with vivid purple color [7]. The promoter substitution or deletion of *BoMYBL2–1* resulted in a purple coloration of cabbage (*Brassica oleracea var. capitata F. rubra*) [12]. Purple leaf genes in ornamental kale and purple stem genes in Chinese kale have been fine mapped [13, 14]. However, it is not reported that a gene or locus controlling purple sepals which are affected by low temperature in broccoli (*Brassica oleracea var. italica*, 2n = 2x = 18).

Some broccoli accessions have purple sepals, and the purple color intensifies during cold weather. Other accessions have green sepals even in cold winter. The purple sepals bring out dull color to flower heads of broccoli. So the broccoli flower heads with green sepals are more welcome and have higher price than those with purple sepals in Chinese market. And breeders tend to breed the broccoli new cultivars which have flower heads with ever-green sepals. It is important to discover the genetic rule, the locus and the gene of the purple sepals for broccoli breeding. We tried to map the locus controlling purple sepals in broccoli by a previous high-dense genetic map which was constructed by referring to TO1000 whole genomic sequences [15]. But it failed to detect a locus. In this study, we mapped the loci controlling the purple sepals by reconstructing a no-reference genetic map based on the previous sequence data submitted to the National Center of Biotechnology Information (NCBI) (the BioProject ID: PRJNA449775) .

## Results

### SLAF markers

SLAF markers were developed based on alignment and clustering of all clean sequence reads using BLAT (BLAST-Like Alignment Tool) software. A total of 182,813 SLAFs were obtained with 61.20-fold average sequencing depth in the maternal line, 65.98-fold in the paternal line, and 15.82-fold in each individual offspring (Table 1). Based on the allele numbers and the sequence differences, SLAF markers could be classified into three types: polymorphic, non-polymorphic and repetitive (Table 2). Of these, 20.77% (37,969 SLAF markers) of the markers were polymorphic. As the segregation group contained DH (doubled haploid) lines, the genotypes that were not aa × bb genotype, and those that lacked parental information were abandoned. This provided us with a high-quality collection of SLAF markers. Together 18,295 SLAF markers out of 37,969 belonged to the aa×bb genotype. Polymorphic SLAF markers with lower-quality or severe partial separation or more than 3 SNPs (single nucleotide polymorphism) or covering less 70% separate individuals were filtered. A final set with 6694 markers was used to construct the genetic map with an average sequencing depth of 81.37-fold in the maternal line, 84-fold in the paternal line, and 15.76-fold in each individual population (Table 1).

**Table 1:** Sequencing depth of specific locus amplified fragment (SLAF) markers

High-quality SLAF markers	
No. of SLAFs	182,813
Average depth in maternal line	61.20×
Average depth in paternal line	65.98×
Average depth in offspring individuals	15.82×
Polymorphic SLAF markers of maps	
No. of SLAFs	6694
Average depth in maternal line	81.37×
Average depth in paternal line	84.00×
Average depth in offspring individuals	15.76×

**Table 2:** Types of specific locus amplified fragment (SLAF) markers identified based on clustering

Type	Polymorphic SLAF	Non-polymorphic SLAF	Repetitive SLAF	Total SLAF
Number	37,969	143,775	1069	182,813
Percentage	20.77%	78.65%	0.58%	100%

Note: Polymorphic SLAF indicates the presence of a polymorphism site in the SLAF tag. The polymorphism site mostly includes SNPs and InDels. Non-polymorphic SLAF refers to the absence of a polymorphic locus in the SLAF tag. Repetitive SLAF refers to SLAF tags located in the repetitive sequence regions. Total SLAF refers to all types of SLAF tags

### Construction of the genetic map

**Fig. 1:**
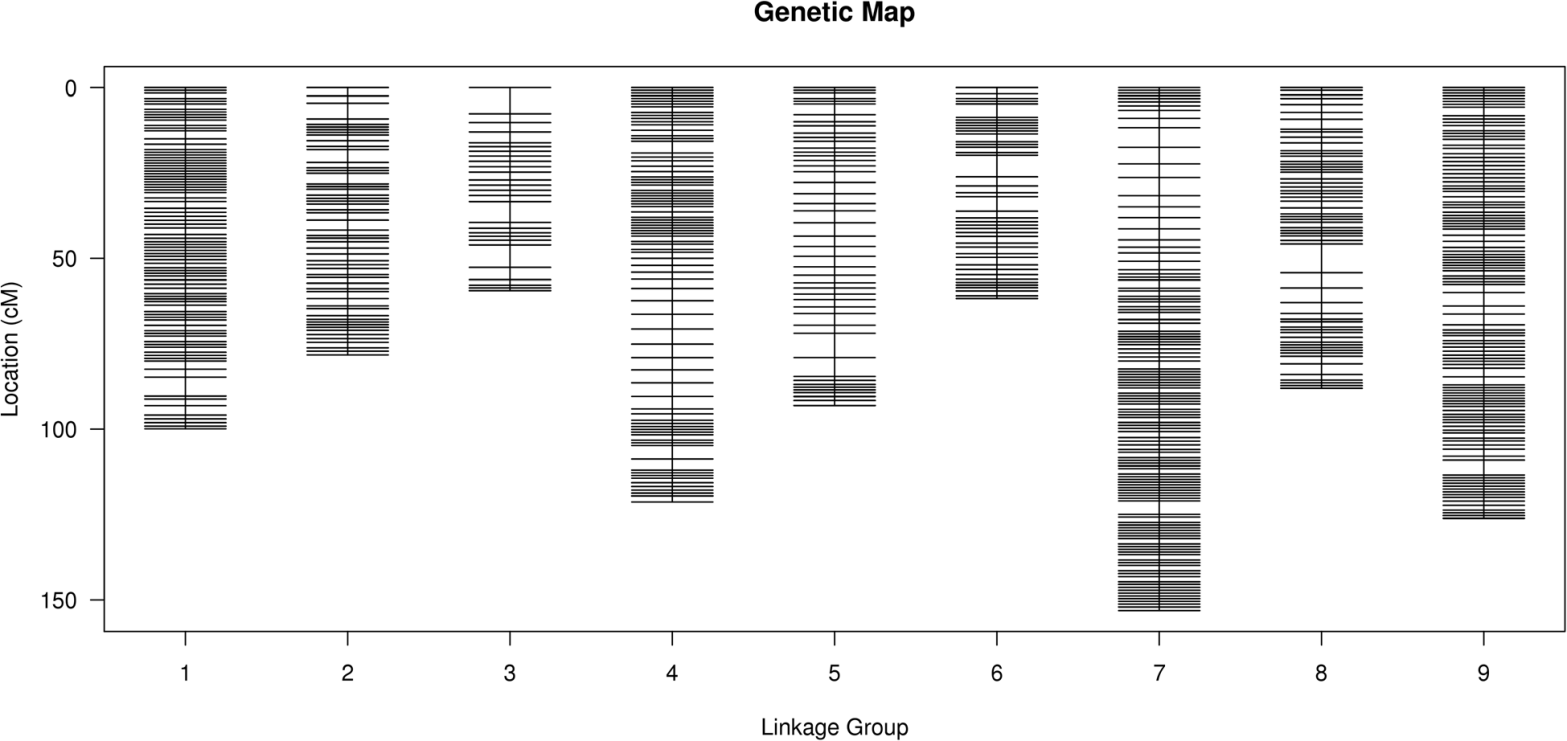
Genetic map of broccoli

A genetic map with 9 linkage groups (LGs) was obtained from 6694 SLAFs containing 12,980 SNPs, with a total genetic distance 880.78 cM, an average genetic distance of 0.16 cM, a maximum gap of 8.41 cM on the LG8. We identified 1358 markers that indicate segregation distortion, with 0.04% of singletons and 23.41% of miss (Fig. 1; Table 3). The individual integrity of the markers in the map was 97.88% (Additional file 1).

**Table 3:** Basic information of the genetic map

LG ID	1	2	3	4	5	6	7	8	9	Total
Total SLAFs	498	922	1539	972	867	469	571	675	181	6694
Total SNPs	961	1963	2745	1949	1625	920	1221	1241	355	12,980
Size (cM)	121.27	99.92	153.12	126.06	78.24	61.7	93.07	88.02	59.38	880.78
Average Distance	0.24	0.11	0.1	0.13	0.09	0.13	0.16	0.13	0.33	0.16
Gaps<=5	1	0.9989	0.9987	1	1	0.9979	0.9965	0.9985	0.9833	0.9971
Max Gap	4.44	5.53	5.75	4.25	4.61	6.33	7.13	8.41	7.63	8.41
Total DS	303	10	276	70	540	16	83	18	42	1358
Singleton(%)	0	0.01	0	0	0	0	0.01	0	0.02	0.04
Miss (%)	2.02	0.58	0.78	0.59	5.66	2.79	1.27	4.36	5.36	23.41

### Collinearity analysis between the genetic map and the reference genome

**Fig. 2:**
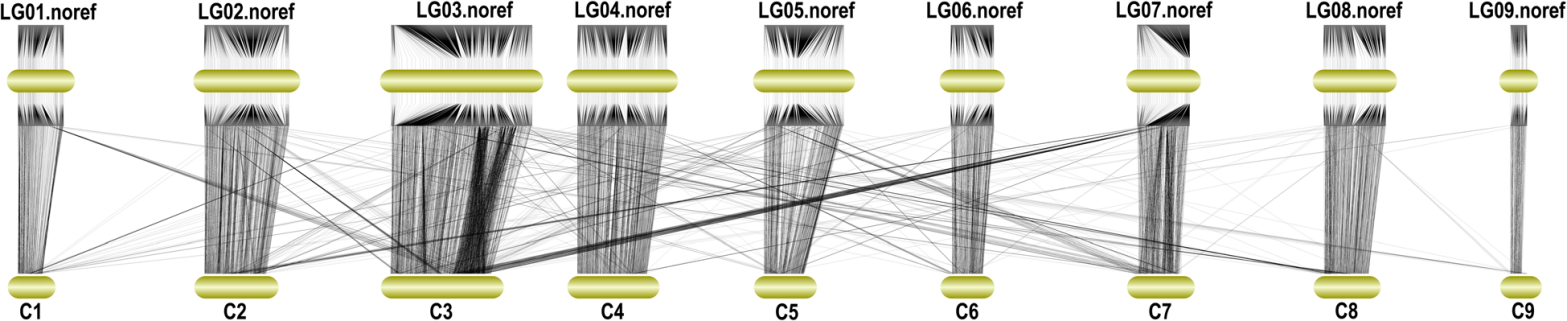
Colinearity between the markers on the genetic map and the reference genome

The sequences of SLAF markers on the map and the sequences of reference genome TO1000 were aligned. Approximately, 95.35% (6383) of the markers from a total of 6694 SLAF markers were mapped to corresponding positions in the reference genome. Only 311 SLAF markers (4.65% of 6694 SLAFs) present in the map could not be assigned to positions in the reference genome. Therefore, the markers in the map show high collinearity to the reference genome (Fig. 2).

### Purple sepal trait of flower head and its inheritance model

**Fig. 3:**
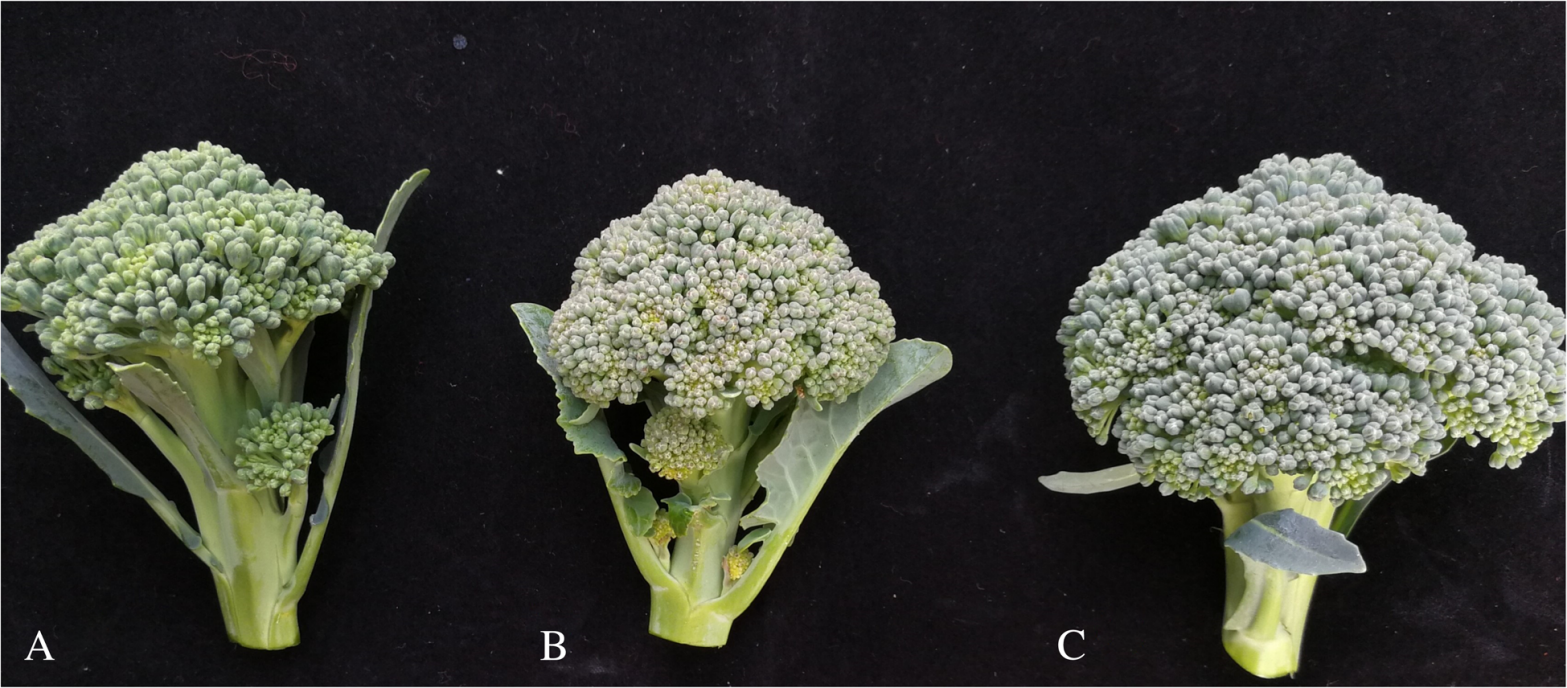
Flower head colors of paternal (**a**), maternal (**b**) lines and hybrid F1 (**c**)

The purple sepal trait of flower heads in the DH population and their parents were surveyed in the autumn of 2015 and 2017 and in the spring of 2018. The sepal color of the maternal line is purple, and low temperature intensifies the purple coloration; while the sepal color of the paternal line never turned purple, even at sub-zero temperatures (Fig. 3). The sepal color of the F_1_ hybrid was purple. Of the 309 genotypes in the F_2_ population, the sepals of 236 individuals were purple, and sepals of 73 were not purple. A χ^2^ test indicated that the fitness of the segregation to the expected ratio of 3:1. From the results, we can infer that the purple sepal trait is likely controlled by a single locus.

### Location of the purple sepal allele

Based on the genetic map and using the phenotype data collected during the autumn of 2015 and 2017, a single locus on LG1 was identified. Both the loci were qPH.C01–1 (30.093–39.543 cM) and qPH.C01–2 (36.393–36.393 cM), respectively. The locus qPH.C01–1 contains qPH.C01–2. That’s, the same single locus region was found based on the data of 2015 and 2017. While three loci, qPH.C01–4 (24.58–24.58 cM), qPH.C01–5 (32.455–32.455 cM), and qPH.C01–3 (36.393–40.331 cM) were found in LG1, based on the data from spring of 2018 (Table 4). These three loci were close to each other in LG1. Apparently, the locus at 36.393 cM on LG1, which accounts for 10.3% of the phenotypic variation, was common in all the three analyses performed.

**Table 4:** Position of the purple sepal allele of flower heads in the two maps

QTL	LG	Range (cM)	LOD	%PVE^a^	ADD^b^	Season^c^
qPH.C01–1	1	30.093–39.543	3.60	16.5	0.19	2014 Aut.
qPH.C01–2	1	36.393–36.393	2.50	10.3	0.16	2017 Aut.
qPH.C01–3	1	36.393–40.331	3.05	10.9	0.16	2018 Spr.
qPH.C01–4	1	24.58–24.58	2.66	9.6	0.15	2018 Spr.
qPH.C01–5	1	32.455–32.455	2.72	9.8	0.15	2018 Spr.

^a^Percentage of the phenotypic variation explained by the *QTL* (Quantitative trait locus); ^b^Additive effect; ^c^time of phenotype survey

## Discussion

Broccoli is one varietas in *B. oleracea* [16]. The genomes of other two varietas in this species, *B. oleracea* var. *capitata* line 02–12 and kale-like TO1000 DH, have been sequenced and successfully assembled [17, 18]. They are very important for genetic research in *B. oleracea* crops, and their genomes have been referenced in the construction of a genetic map in cauliflower, and for fine mapping in ornamental kale and Chinese kale [13, 14, 19–21]. However, there are assembly errors and mis-anchored sequence scaffolds in the cabbage genome ‘02–12’ and kale-like genome TO1000 [14, 22]. Actually, such gaps and misassemblies are common in whole-genome assembly of many species [13, 22–24]. Biological complexities, especially the Brassicaceae-specific triplication events and continuous amplification of transposable elements in *B. oleracea*, have complicated the assembly of the genomes, resulting in gaps and misassemblies [25–27]. These errors lead to incorrect mapping, failure to accurately identify and clone gene(s) based on a related genetic map, inability to identify a related locus, and inefficient marker-assisted selection [13, 14]. Moreover, one or even several genotypes sequenced at a high resolution may not contain all the sequences for that particular species. For example, there were huge structural variations including inversions, translocations, and presence/absence variations between the two genomes of elite ‘indica’ rice varieties’ ZS97RS1 and MH63RS1. Besides, there were some genes present in variety ZS97RS1 that were absent in MH63RS1, and vice-versa [28]. In our previous study, no locus related to the purple sepal trait was found in that genetic mapping exercise, which was constructed based on the reference genome TO1000. Broccoli and the kale-like line TO1000, between which the main phenotypic difference is that the former has a big flower head, are two different varietas of *B. oleracea*. It is possible that sequences differences and structural variations exist between the two varietas, making the identification of the purple sepal allele in the genetic map difficult, particularly, if based on the TO1000 reference genome. So, it was necessary to reconstruct a non-reference genetic map for mapping the purple sepal loci.

One locus related to the purple sepal trait on the map, which was located at 36.393 cM in LG1 was identified based on the phenotypic data collected during two autumn seasons, while the three loci located at intervals of 15.751 cM, 24.58 cM, and 40.331 cM from LG1, were found based on the phenotypic data from the spring of 2018. The identification of three loci seems inconsistent with the results from the χ^2^ test, which, indicates that the purple sepal trait may be controlled by a single locus. As the purple sepal color in the maternal line intensifies during low temperature, potential regulatory genes related to the purple color could be induced by cold. In addition, the proximity of all the three loci in LG1, may indicate the presence of a regulatory network involved in controlling the purple coloration, and perhaps is active and sensitive to low temperature, yet the related trait is difficult for the human eye to visualize. In autumn, the temperature reaches 30 °C, coinciding with the beginning of growth, after which the temperature decreases. The temperature during the whole growth period stays above zero. In contrast, the temperature during spring increases gradually and reaches a high point. Assuming that the purple color is regulated by low temperature, it may be easier to observe the purple sepal color of broccoli flower heads in spring. These two loci could be related to low temperature inducing purple coloration of sepals in the flower heads of broccoli. So the purple sepal might be controlled by a single major locus and two minor loci, which would be confirmed by fine mapping, for the other both loci had low LOD values and low phenotypic variation explained by the QTLs.

Anthocyanins are catalytically synthesized by a series of enzymes encoded by corresponding genes, such as chalcone synthase (*CHS*), chalcone isomerase (*CHI*), flavanone 3-hydroxylase (*F3H*), flavanone 3′-hydroxylase (*F3’H*), flavonol synthase (*FLS*), dihydroflavonol 4-reductase (*DFR*), encoding glutathione-*S*-transferases (*GST*), leucoanthocyaniidin oxgenase (*LDOX*), anthocyanidin reductase (*ANR*), UDP-glucose: flavonoid 3-O-glucosyltransferase (*UD3GT*) [12, 29, 30]. While, anthocyanin biosynthesis also is regulated by transcription factors such as MYB, bHLH, WD40, and ZAT6 [7, 30–33]. Anthocyanins biosynthesis and regulation not only involve many structural genes and transcription factors, but also are influenced by environmental factors, such as light, temperature and other substances [12, 34–36]. The expression of structural genes of anthocyanins biosynthesis such as *CHS, DFR,* and *GST* were higher in low temperature conditions [37–39]. Light could induce the expression of the transcription factor genes (such as *MYB75* and *MYB90*), the structural genes (such as *CHS, DFR, F3H,* and *LDOX*), the kinase gene (such as *MAP KINASE4*) and ethylene response factor genes which are involved in anthocyanin biosynthesis [40–42]. A PA1-type MYB transcription factor, MdMYBPA1 was identified from red-fleshed apple and it redirected the flavonoid biosynthetic pathway by its promoter’s low-temperature-responsive (LTR) cis-element directly binging MdbHLH33 in low temperature conditions [43]. In purple cabbage, a MYB transcript factors, BoMYBL2–1, negatively regulated anthocyanin synthesis [12]. Substitution or deletion of its promoter resulted in different degree of purple coloration and different sensitivity to low temperature [12]. A R2R3 MYB transcription factor, *BoPAP1*, might be responsible for purple leaves and up regulated by low temperature in purple kale [10]. These researches show that different plants have different molecular regulation mechanism in anthocyanins accumulation induced by low temperature. Anthocyanins are obviously accumulated in leaves and induced by low temperature in the cabbage and the kale [10, 12]. In this study, no obvious purple color are observed in leaves of the broccoli line DH16–2, but its sepals are purple and become deep purple in cold conditions. What’s more, the genes for the purple traits in kale, cabbage and kohlrabi were all mapped to the chromosome 6 which was the same locus as the *BoMYB2* gene in cauliflower [44]. While the loci controlling the purple sepals in broccoli was located on the LG1 in this study. It is still unknown which genes are responsible for that. Further study will be exerted for better understanding of anthyocanins biosynthesis and regulation in purple sepals of broccoli.

## Conclusions

In this study, we constructed a high-density genetic map using a DH segregation population of a cross between DH16–2 and DH28–4. Based on this map, a major locus and two minor loci of the purple sepal using phenotypic data collected from three seasons was detected on LG1.

## Methods

### Plant materials

The maternal line DH16–2 (purple sepals), paternal line DH28–4 (green sepals), their hybrid and the segregating population with 127 DH lines was generated using the microspore culturing methods. The parental lines and segregating population individuals were planted in the Yangdu Experimental Greenhouse of the Zhejiang Academy of Agricultural Sciences in August 2014, August 2017 and January 2018. The purple sepal trait was surveyed when the flower heads were mature during the crop season. During the period of cultivation, every plant was checked to make sure no leave covering on the flower head in order to avoid the effect of light on the sepals color.

### SLAF markers and genotyping

Based on the raw data from BioProject (PRJNA449775), SLAF markers were identified, and genotyping was performed as described in [45]. Initially, all the low-quality reads were removed and high-quality raw reads were sorted to each progeny according to the duplex barcodes. Clean reads were then clustered based on similarity over 90% after the barcodes and the terminal 5 bp positions were trimmed from each high-quality read. The sequences that were clustered together were defined as one SLAF locus [46]. SNP loci for each SLAF locus was identified between the parents. SLAF markers with more than 3 SNPs were deleted. Alleles of every SLAF locus were recognized on the basis of the reads from the parents. Diploid broccoli plants were used in this study, so, each SLAF locus could include a maximum of 4 genotypes. Only SLAF loci with two to four alleles were reserved as potential markers and those SLAF loci with more than 4 alleles were categorized as repetitive SLAF markers and rejected. All polymorphic SLAF marker loci consistent both, in the parental and offspring SNP loci were genotyped. The marker codes of the polymorphic SLAF markers were analyzed on the basis of the population type, such as DH with only one segregation type (aa × bb).

Genotype scoring was done using a Bayesian approach to guarantee consistent genotyping quality [45]. First, a posterior conditional probability was calculated based on the coverage of each allele and the number of SNPs. Genotyping quality score based on the probability was used to select qualified markers for the succeeding analysis. Low-quality markers or individuals were discarded in the dynamic process. When the average genotype quality values of all SLAF markers reached the critical value, the process stopped. High-quality SLAF markers for constructing the genetic map were screened using the following criteria: Sequences with more than 10-fold depth of the parents, markers covering more than 70% genotypes of all offspring, and the segregation distortion as examined by the χ^2^ test.

### Genetic map construction and QTL mapping

The HighMap software developed by Beijing Biomarker Technologies Corporation, was utilized to construct a high-density and high-quality map [47]. Recombinant frequencies and the maximum likelihood method (MLOD) scores between markers were used to deduce the linkage phases and two-point analysis was used. Molecular markers were divided into different linkage groups based on the MLOD score, and each linkage group was regarded as a chromosome. A genetic map was preliminarily constructed, and the initial sequence of the markers was obtained based on the MLOD score. As the genotyping results contain certain errors related to the molecular markers, genotype correction was performed based on the sequence of the markers in the map. A high-quality genetic map was finally constructed using the Kosambi mapping function after several rounds of correction [48]. MapQTL 5.0 was used to analyze the phenotypic data for interval mapping.

### Comparison between the genetic map and the reference genome

In order to compare the linearity between markers on the genetic map and the reference genome, SLAF markers sequences of the map were positioned on the reference genome, kale-like line TO1000. The physical locations of the SLAF markers in the map were identified. These physical locations in the map were compared to their positions in the reference genome and a linear analysis was conducted.

## Additional file

Additional file 1: Individual integrity of specific locus amplified fragment (SLAF) markers in the genetic map. (PNG 15 kb)

## Abbreviations

DH: Doubled haploid
LG: Linkage group
QTL: Quantitative trail locus
SLAF: Specific locus amplified fragment
