# Supplementary figures and images for: Construction of a high-density genetic map and identification of loci controlling purple sepal trait of flower head in Brassica oleracea L. italica
Source: BMC Plant Biol. 2019 May 30;19:228. doi: 10.1186/s12870-019-1831-x (PMC6543578; doi:10.1186/s12870-019-1831-x)

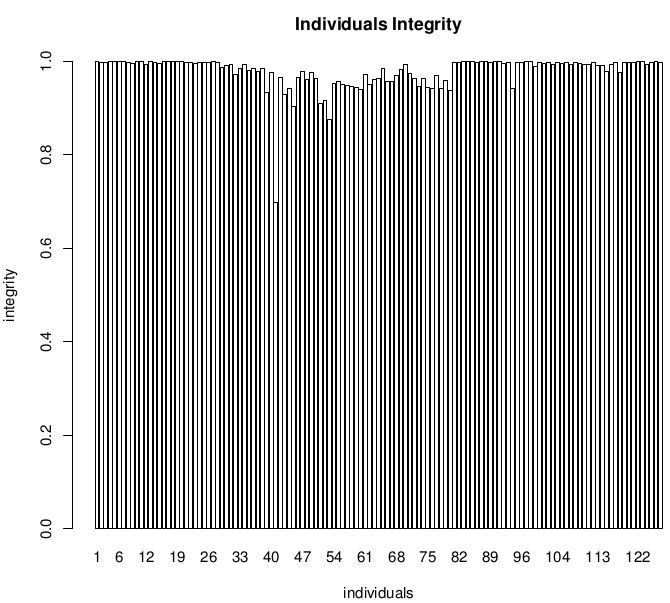

Supplement: Supplementary file 1 — Individual integrity of specific locus amplified fragment (SLAF) markers in the genetic map. (PNG 15 kb) [file 12870_2019_1831_MOESM1_ESM.png]
